# Traditional foraging for ecological transition? Wild food ethnobotany among three ethnic groups in the highlands of the eastern Hindukush, North Pakistan

**DOI:** 10.1186/s13002-023-00581-9

**Published:** 2023-03-31

**Authors:** Amir Hasan Khan, Muhammad Adil, Muhammad Abdul Aziz, Renata Sõukand, Andrea Pieroni

**Affiliations:** 1 Qurtuba University of Science and Information Technology D.I Khan,, Peshawar Khyber, Pakhtunkhwa Pakistan; 2 Department of Environmental Sciences, Informatics and Statistics, Ca’ Foscari University of Venice, Via Torino 155, 30172 Venice, Italy; 3 University of Gastronomic Sciences, Piazza Vittorio Emanuele II 9, 12042 Pollenzo, Bra, Italy; 4 Department of Medical Analysis, Tishk International University, Erbil, 4401 Kurdistan Iraq

**Keywords:** Ethnobotany, Food heritage, Local ecological knowledge, Wild food plants, Pakistan

## Abstract

**Background:**

The Patrak Valley is home to communities, which have been inextricably linked with nature for generations, and local plant knowledge (LPK) represents an important part of their local cultural diversity. In general, globalization has come at the expense of local plant knowledge among several mountain societies, and therefore the current investigation has been undertaken to record the (possibly) last remaining wild food plant/mushroom foraging practices among Pathans, Kohistanis, and Gujjars living in the highlands of the Hindukush, North Pakistan.

**Methods:**

Data on the uses of wild food plants and mushrooms (WFPs) were collected through 120 semi-structured interviews. The data were cross-culturally compared among the three linguistic groups. Venn diagrams were used to visualize the comparative analysis. To determine the patterns of similarities in plant use among the different ethnic groups, we used the Jaccard similarity index (JI). The recorded data were also compared with the existing Pakistani food ethnobotanical literature.

**Results:**

A total of 68 WFPs were recorded, the majority of which were used as raw snacks and as cooked vegetables. Fruit was the most frequently reported plant part among the three researched groups. Cross-cultural comparison revealed that 37% of the used plants were commonly shared by the three studied groups. Pathans have retained rich knowledge on WFPs, and they show a comparatively closer affinity with Kohistanis is the use of WFPs compared to Gujjars. While we observed some idiosyncrasies for each of the researched groups, the distinctive plant uses among Gujjars provide insight into their food ecology, their particular human–ecological system centered on mobile pastoralism and their limited exchanges of local food/ecological knowledge due to endogamic patterns. A literature survey revealed some novel or little-known ingredients within Pakistani food ethnobotany/ethnomycology, such as *Aesculus indica*, *Agaricus campestris*, *Apteranthes tuberculata*, *Duchesnea indica*, *Equisetum arvense*, *Eremurus himalaicus*, *Isodon rugosus*, *Morella esculenta*, *Sophora mollis*, and *Drimia indica*.

**Conclusion:**

The researched communities have retained important plant knowledge which could be implemented through future development programs considering that most of these traditional foraging practices fulfill environmental and social sustainability standards. Further field studies are required to thoroughly investigate the patterns of foraging among highland pastoral societies in other parts of the Hindukush region and especially their potential for the ongoing ecological transition.

## Background

Given the dreadful consequences of the ongoing climate change on local ecologies across the globe, recently world leaders have agreed on new measures in order to tackle the negative impacts of environmental disasters and help vulnerable societies. In particular, food and agricultural policies need to be reshaped in order to not only prevent unsustainable ecological practices but also undo the unjust impacts of globalized economies on vulnerable societies in certain geopolitical contexts. Wild food mushrooms and plants (WFPs) and related knowledge, therefore, represent rich grounds for local communities to realize their food sovereignty, and applied ethnobotany could empower marginalized societies and facilitate their sustainable food use of commonly available natural resources.

The highlands of the Hindukush Mountains in North Pakistan are home to peripheral communities, for which mobile pastoralism is often a crucial part of their social and cultural identities. Mobile pastoralism also provides practical opportunities to continuously encounter different socio-ecological landscapes [1–3], which in turn allows these communities to constantly reshape their human–ecological relationships and to engage in intercultural interactions and create social networks with other cultural groups [4–7]. The inextricable link between pastoralist societies and plant use has been investigated in many studies across the globe [for example 8–9], but in Pakistan it has been little explored and only a few investigations [10] have been carried out on the subject. In the extreme north of the Pakistani Hindukush, we observed that local plant knowledge (LPK) has been crucial in sustaining traditional food systems and historically this knowledge, which is a cultural entity, has been renegotiated within a multicultural environment [11]. Therefore, these findings are highly pertinent to exploring the phenomenon of the exchange of LPK and its renegotiation within multicultural societies in other parts of the mountainous region. In this regard, the Hindukush is an important “*cultural area*” [12] and represents a valuable arena for human ecological studies.

It has been argued that mountain territories sometimes present favorable environments for certain societies to keep their local plant knowledge distinct from other groups. However, some research findings challenge this idea [13, 14] and thus the question remains: do mountains function as a refuge for preserving the idiosyncrasy of plant use among a particular ethnic group or not?

We should keep in mind that remoteness alone does not guarantee the distinctiveness of LPK, as other factors such as strong sociocultural negotiations play a role in this regard [11, 15].

The current study explored traditional WFP foraging practices among three different ethnolinguistic groups, i.e., Gujjars, Kohistanis, and Pathans, living in the Patrak Valley in the Eastern Hindukush region of Pakistan, especially from the standpoint of providing concrete baseline data to further facilitate ecological transition, e.g., the holistic sustainability of food systems among the local communities. We aimed to investigate cross-cultural WFP use and to possibly determine the diffusion of these three food ethnobotanies. It is worth mentioning that among the three ethnic groups, Gujjars have a very distinctive lifestyle, as they frequently practice mobile pastoralism [16] and their summer pastures known as “*charagah*'' are important sites for collecting WFPs [17].

The current study is therefore a timely attempt to investigate foraging patterns among the aforementioned groups, which could represent an important addition to the ethnobiological literature of Pakistan. The study may also improve the understanding of the human–nature relationship in this remote mountain valley and could help policy makers obtain incentives for helping in future food sovereignty-centered strategies, as these high mountain communities are extremely vulnerable to food insecurity and local resource management has been greatly challenged by modernization [18, 19] and recent socioeconomic transformations [6, 20–23].

The specific objectives of the research were:

a. to record local knowledge on WFPs among the three considered ethnolinguistic groups,

b. to cross-culturally compare local plant use among the three groups,

c. to propose recommendations in order to assure future food security, sustainable food systems, and socio-ecological resilience among marginalized mountain societies.

## Methods

### Study area and the studied communities

**Fig. 1:**
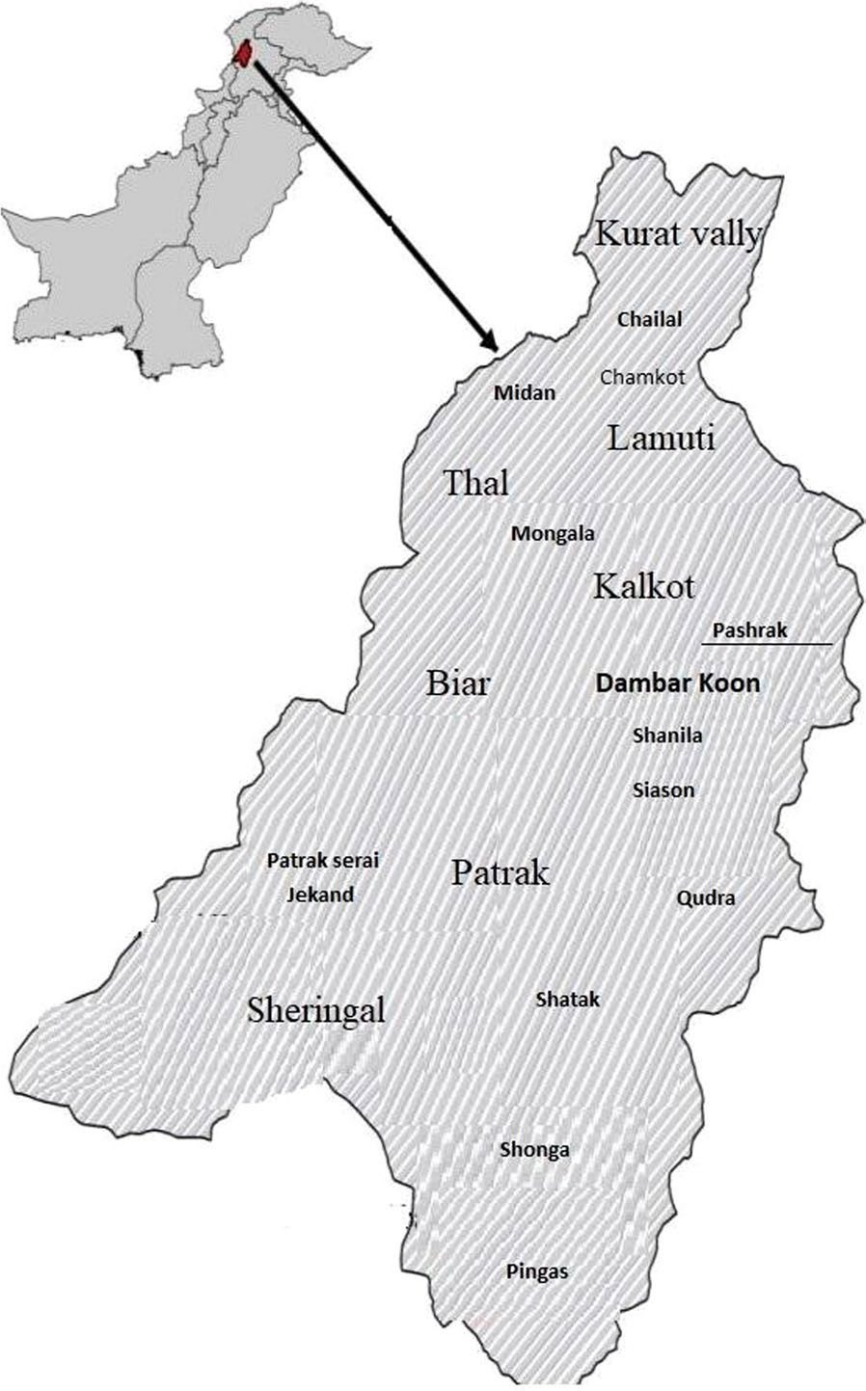
Map of the study area

The Patrak Valley is located in the Upper Dir District of Khyber Pakhtunkhwa (KPK), Pakistan (Fig. 1). It is located at 35.3440° north latitude and 72.0590° east longitude. The study area is part of the Hindukush Mountain Range. The area is populated by Pathans, Kohistanis, and Gujjars. The former two groups live in valley-bottom villages, while Gujjars inhabit high elevation pastures and frequently migrate to other areas in winter. The characteristics of the studied communities are shown in Table 1. Pathans are the dominant cultural group followed by Kohistanis, while Gujjars are recognized as a minority group in the valley. Historically, these communities have been pastoralists, although in more recent times societal transition has resulted in many Pathans and Kohistanis adopting a different lifestyle to that which they inherited from their ancestors. However, Gujjars have retained their traditional lifestyle and regularly practice mobile pastoralism in the highlands of the mountain range. Gujjars spend the summer along with their herd in highland pastures and during winter they come down the mountain with a few households staying in the lower valley, while others spend the winter in Timergara, Charsadda, Chakdara, and Nowshera.

**Table 1:** Characteristics of the villages and studied communities

Language	Village	Elevation (Meters Above Sea Level)	Number of Households	Number of Interviewees (Male/Female)	Number of study participants	Endogamic/Exogamic Rule	Mean age of the study participants	Estimated average socio‐economic status of the study participants	Main occupation
Pashto	Dambar Koon	1950	219	5 males	40	Exogamic	69	Middle	Farming
				2 females					
	Shanila	1895	94	6 males					
				1 female					
	Siason	2010	284	5 males					
				0 females					
	Patrak serai	2145	350	5 males					
				2 females					
	Jekand	2001	240	6 males					
				1 female					
	Dogal	1830	280	5 males					
				2 females					
Kohistani	Chailal	1923	93	6 males	40	Exogamic	65	Middle	Farming and pastoralism
				1 female					
	Midan	2120	95	5 males					
				3 females					
	Mongala	2009	131	8 males					
				2 females					
	Pashrak	2210	169	4 males					
				3 females					
	Patrak	2191	616	6 males					
				2 females					
Gujri	Chamkot	2177	186	3 males	40	Endogamic	67	Low	Farming only in summer and pastoralism
				1 female					
	Chakar Batala	2305	99	4 males					
				3 females					
	Gujar Lala	1820	33	4 males					
				2 females					
	Qudra	2140	208	6 males					
				1 females					
	Shatak	1966	224	5 males					
				0 females					
	Shonga	2155	195	4 males					
				1 females					
	Pingas	2056	212	4 males					
				2 females					

### Climate and vegetation

**Fig. 2:**
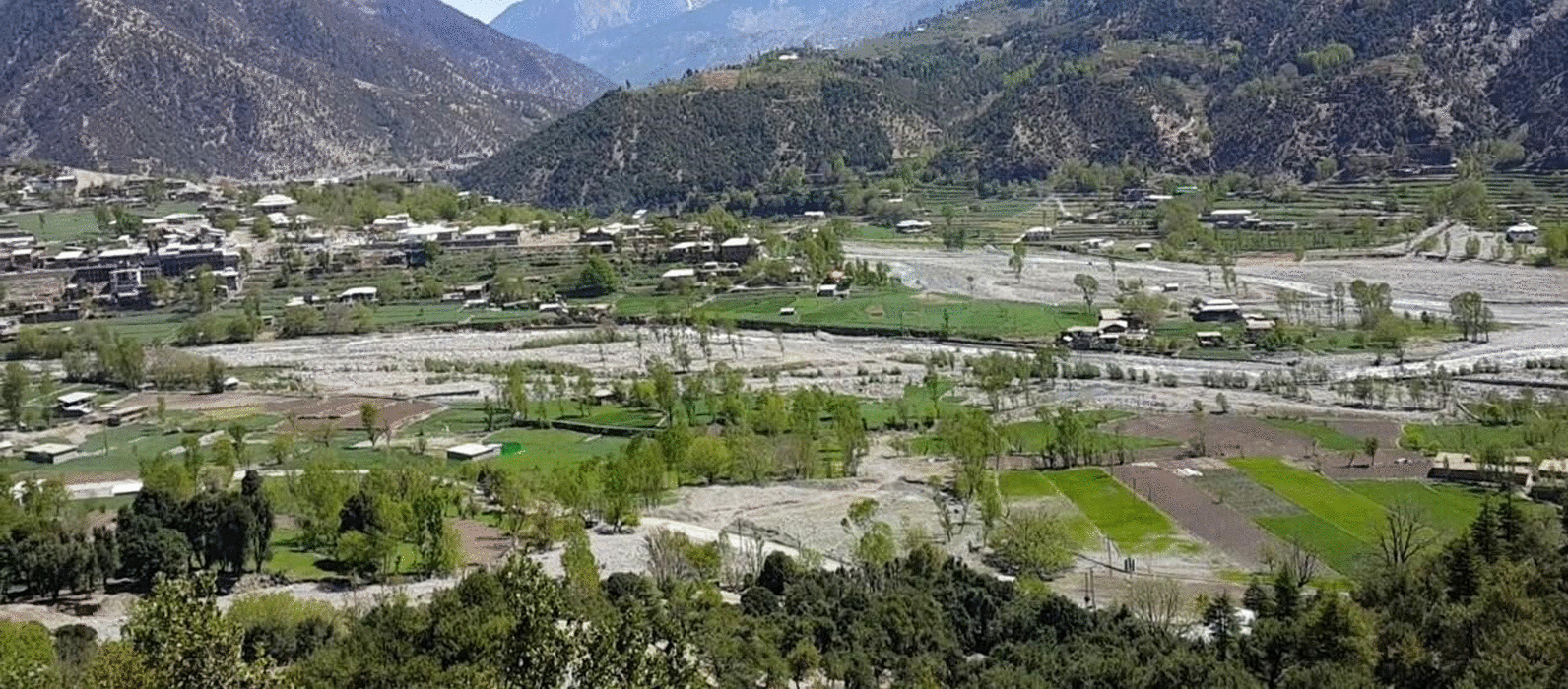
The landscape of the Patrak Valley

The Patrak Valley, which is part of Dir Kohistan, has four distinct seasons, i.e., summer, winter, spring, and autumn. The landscape of the area is shown in Fig. 2. The area is located at an elevation of 2,111 m a.s.l. (above sea level). The winter season is very cold, and as a result, a large number of people residing in the upper parts of the region migrate to lower areas along with their livestock. In winter, the valley is covered with snow for four months or more. A minimum temperature of -7 °C has been recorded in winter. Average rainfall in the month of March is approximately 269.6 mm.

The forests of Dir Kohistan Valley can be broadly categorized into the following types: 1. Shrub Oak forests; 2. Pure Deodar forests; 3. Mixed Deodar, Kail, Fir, and Spruce forests; 4. Mixed Fir and Spruce forests; and 5. Alpine pastures. Shrub and oak forests exist in lower areas of the valley ranging from 1,600 to 1,800 m a.s.l. These trees are under heavy pressure from cutting as the wood is used for fuel. Coniferous forests grow at altitudes ranging from 3,000 to 6,000 m a.s.l. which is upper limit of tree growth. Above the tree line, alpine pastures cover the whole area. Common tree species include *Pinus roxburghii*, *Pinus wallichiana*, *Abies pindrow*, *Picea smithiana*, *Taxus baccatta*, *Viburnum grandiflorum*, *Quercus baloot*, *Olea ferruginea,* and *Morus alba*.

### Ethnobotanical survey

A field survey was conducted from July to August 2022 in the Patrak Valley of the Hindukush. Data were gathered through semi-structured interviews with the elderly community members among the three studied groups. Forty participants were sampled from each of the ethnolinguistic groups. Participants were selected through a pseudo-random sampling technique (pseudorandomness in sampling measures the extent to which a sequence of number of participants, though produced by a completely deterministic and repeatable process, appear to be patternless) among each of the linguistic groups, as we contend that food ethnobotanical knowledge is an element of daily use among the local communities and it does not require any special expertise to be shared since food is consumed by every individual in any given society. Prior verbal consent was obtained from each participant in order to record the reported information and ethnobotanical data. Before conducting in-depth interviews with the participants, we explained the main objectives of the study. We asked various questions regarding the use of WFPs. We recorded the WFP uses mentioned by the study participants along with their botanical names, local names, parts used, methods of culinary preparation, and frequency of quotation. We also collected information on the gathering locations of WFPs. All interviews were conducted in the participant’s native language with the help of a local translator. We used open-ended questions and also recorded some qualitative data through direct observation. The interviews and discussions were mainly focused on gathered WFPs that were used as raw snacks, as cooked vegetables, in seasonings, and for recreational teas. Moreover, some specific questions were asked about the uses of WFPs in lacto-fermentation and dairy products. During the ethnobotanical field survey, we strictly followed the recommendations of the International Society of Ethnobiology [24]. We received approval to photograph the informants and the local dishes. We gratefully acknowledge the cooperation of the selected informants who took part in the semi-structured interviews, without whom it would not have been possible to conduct this study. At the end of the survey, we also collected voucher specimens for each of the quoted plants. The plants were identified by a taxonomist at the Department of Botany, University of Malakand, Pakistan, with the help of *Flora of Pakistan* [25–28]. Voucher specimens were deposited in the Herbarium of Qurtuba University, Peshawar. The world Flora Online [29] was used to verify the nomenclature and classification of each plant taxon, and the Index Fungorum [30] was utilized to crosscheck fungal nomenclature. The ethnobotanical data were presented along with their botanical families and voucher specimen numbers. We have also given common English names to each of the quoted plant taxa. It should be noted that all the English names for the given plants are available on Wikipedia (https://www.wikipedia.org/) except a few ones that we have taken from other online sources such as Flowers of India (https://flowersofindia.net/) and India Biodiversity Portal (https://indiabiodiversity.org/species/show/243959).

### Data analysis

For each of the reported plant taxa, use reports were counted. The data were organized in MS Excel. We tabulated three different data sets, with each set representing one of the studied ethnolinguistic groups. We cross-culturally compared the different data sets, which we then visualized through Venn diagrams. For determining the similarities in plant use among the different data sets, we used the Jaccard similarity index (JI), which was calculated as:

$$J\left( {X,Y} \right) = \left| {X \cap Y} \right|/\left| {X \cup Y} \right|$$

where X = individual set of plant usage documented by group X, and Y = individual set of plant usage documented by group Y.

Moreover, we qualitatively compared the current data with other studies on WFPs carried out in Pakistan [10, 11, 15, 17, 31–38].

## Results and discussion

### Reported wild food plants and their uses

**Fig. 3:**
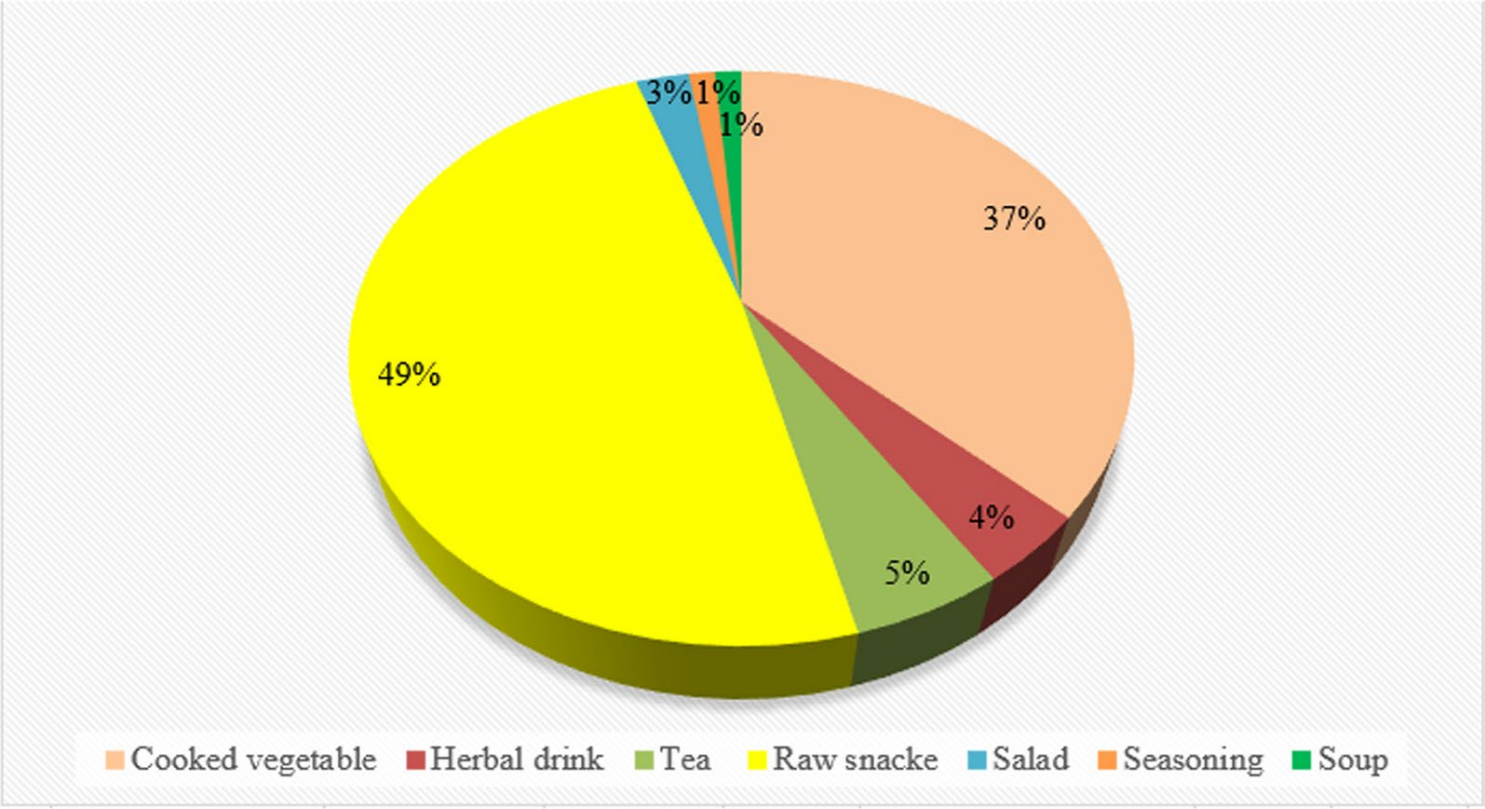
Methods of preparation of the wild food plants used in the study area

A total of 68 taxa were documented among the three studied groups. The majority of the plants were consumed as raw snacks (37 taxa, 49%) and as cooked vegetables (28 taxa, 37%), while a few plants were utilized in seasonings and for recreational herbal teas (1%) (Table 2). Seven distinct types of food preparations were recorded: cooked vegetables, chutneys (a family of hot, sour, and sour-spicy condiments and sauces typical of South Asian cuisines), herbal drinks (plant material infused in cold water), herbal teas (plant material infused in hot water), salads (raw plants consumed at the table as appetizers or in combination with other food items), raw snacks, and seasonings/spices (Fig. 3).

**Table 2:** Gathered WFPs recorded among the studied communities in Dir Kohistan; P: Pashto, G: Gujjar, K: Kohistani

Botanical Taxon; Family; Botanical Voucher Specimen Code	Recorded Local Name	English or common name	Parts Used	Recorded Local Food Uses	Pathans	Kohistanis	Gujjars
*Aesculus indica* (Wall. ex Cambess.) Hook; Sapindaceae; QURTUBA 0044	Satal ^K^	Indian horse-chestnut	Seeds	Raw snack ^K^	**–**	11	**–**
*Ziziphus jujuba* Mill.; Rhamnaceae; QURTUBA 0045	Markhanray ^P^	Common jujube	Fruits	Raw snack ^G^	30	**–**	26
	Sengri ^G^						
*Allium carolinianum* Redouté; Amaryllidaceae; QURTUBA 0046	Ogai ^P^	Wild onion	Leaves	Salad ^P^	4	**–**	**–**
*Berberis lycium* Royle; Berberidaceae; QURTUBA 0047	Iees ^K^	Indian barberry	Fruits	Raw snack ^K, P, G^	13	17	6
	Kwaray ^P^						
	Sumro ^G^						
*Apteranthes tuberculata* (N.E.Br.) Meve & Liede.; Apocynaceae.; QURTUBA 0048	Pamankay ^P^	Chongan	Aerial parts	Cooked ^P^	25	**–**	**–**
*Carthamus oxyacantha* M.Bieb.; Asteraceae; QURTUBA 0049	Mulachu/zeer ^K^	Wild safflower	Seeds	Cooked ^K^	**–**	13	**–**
	Jero ^G^			Tea ^G^			
*Celtis caucasica* Willd.; Cannabaceae; QURTUBA 0050	Boboo ^K^	Caucasian hackberry	Fruits	Raw snack ^K, P, G^	26	16	19
	Taghaga ^P^						
	But karan ^G^						
*Chenopodium album* L.; Amaranthaceae; QURTUBA 0051	Sarmi ^K^	Bacon weed, Fat hen, Goosefoot, Pigweed, White goosefoot	Aerial parts	Cooked ^K, P, G^	23	14	11
	Sarmay ^P^						
	Batun ^G^						
*Chenopodium murale* L.; Amaranthaceae; QURTUBA 0052	Gunri ^K^	Nettle-leaved goosefoot	Aerial parts	Cooked ^K, P, G^	27	10	18
	Churlai ^P^						
	Chuaie ^G^						
*Cichorium intybus* L.; Asteraceae; QURTUBA 0053	Shamakay ^P^	Blue sailors, Chicory, Coffee weed, Common chicory, Cornflower, Italian dandelion, Succory	Aerial parts	Cooked ^K, P, G^	15	7	17
	Found kash ^K^						
	Shamokeo ^G^						
*Cotoneaster nummularius* Fisch. & C.A.Mey; Rosaceae; QURTUBA 0054	Udhundor ^K^	Nummular or coinwort cotoneaster	Fruits	Cooked ^K^	6	11	3
	Kharawa ^P^			Raw snack ^P^			
	Masloor ^G^			Cooked ^G^			
*Dysphania botrys* (L.) Mosyakin & Clemants; Amaranthaceae; QURTUBA 0055	Khurpen ^K^	Jerusalem oak goosefoot	Aerial parts	Cooked ^K, P, G^	20	9	15
	Kharawa ^P^						
	Lowar ^G^						
*Diospyros lotus* L.; Ebenaceae; QURTUBA 0056	Keshen amalok ^K^	Date-plum	Fruits	Raw snack ^K, P, G^	28	23	29
	Toor amlok ^P^						
	Kawo amlok ^G^						
*Duchesnea indica* (Andrews) Teschem; Rosaceae; QURTUBA 0057	Mayako ^G^	Indian strawberry	Fruits	Raw snack ^G^	**–**	**–**	5
*Equisetum arvense* L.; Equisetaceae; QURTUBA 0058	Darhabro ^G^	Horsetail	Aerial parts	Cooked ^G^	–	**–**	12
*Eremurus himalaicus* Baker; Asphodelaceae; QURTUBA 0059	Shella ^P^	Foxtail lily	Aerial parts	Cooked^p^	29	**–**	**–**
*Eruca vesicaria* (L.) Cav.; Brassicaceae; QURTUBA 0060	Laken ^K^	Arugula or rocket	Aerial parts	Cooked ^K, P^	4	14	**–**
	Jamama ^P^						
*Ficus palmata* Forssk.; Moraceae; QURTUBA 0061	Sarmangol ^K^	Punjab fig	Fruits	Raw snack ^K, P^	29	24	**–**
	Inzar ^P^						
*Hypericum perforatum* L.; Hypericaceae; QURTUBA 0062	Shen chai ^P^	St. John's wort	Leaves	Tea ^P, K^	17	12	**–**
	Fotarga ^K^						
*Isodon rugosus* (Wall.) Codd.; Lamiaceae; QURTUBA 0063	Salool ^K^	Wrinkled Leaf Isodon	Aerial parts	Herbal drink ^K, P, G^	21	12	16
	Krachay ^P^						
	Kurkuri ^G^						
*Juglan regia* L.; Juglandaceae; QURTUBA 0064	Chur ^K^	English walnut	Fruits	Raw snack ^K, P, G^	30	30	30
	Ghuz ^P^						
	Akhori ^G^						
*Lactuca sativa* L.; Asteraceae; QURTUBA 0065	Salad ^P^	Lettuce	Aerial parts	Raw snack ^P^	13	**–**	**–**
*Lathyrus aphaca* L.; Fabaceae; QURTUBA 0066	Kurkaman ^P^	Yellow pea	Roots	Cooked ^P^	23	**–**	**–**
*Malva neglecta* Wallr Malvaceae; QURTUBA 0067	Sechal sha ^K^	Common mallow	Aerial parts	Cooked ^K, P, G^	24	15	10
	Panerak ^P^						
	Sochar ^G^						
*Malva sylvestris* L.; Malvaceae; QURTUBA 0068	Samchal ^P^	Common mallow	Aerial parts	Cooked ^P, G^	8	**–**	6
	Suchanr ^G^						
*Marsilea quadrifolia* L.; Marsileaceae; QURTUBA 0069	Chapatray ^P^	Water clover	Aerial parts	Cooked ^K, P, G^	12	16	22
	Chuka chook ^G^						
	Chapatri ^K^						
*Medicago polymorpha* L.; Fabaceae; QURTUBA 0070	Speshtaray ^P^	Toothed bur clover	Aerial parts	Cooked ^P^	25	**–**	**–**
*Mentha longifolia* (L.) L.; Lamiaceae; QURTUBA 0071	Dhoop ^K^	Horse mint	Aerial parts	Raw snack ^K^	20	18	26
	Wenalay ^P^			Herbal drink ^P^			
	Wenlo ^G^			Raw snack ^G^			
*Mentha spicata* L.; Lamiaceae; QURTUBA 0072	Podeno ^K, G^	Spearmint	Leaves	Tea ^K^	25	15	21
	Podina ^P^			Salad ^G, P^			
*Ziziphus oxyphylla* Edgew.; *Rhamnaceae*; *QURTUBA 0110*	Enalai ^P^	Pointed-leaf jujube	Fruits	Raw snack ^P^	20	**–**	**–**
*Morus alba* L.; Moraceae; QURTUBA 0074	Spen toth ^P^	White mulberry	Fruits	Raw snack ^P^	24	**–**	**–**
*Morus macroura* var*. laxiflora* G.K.Upadhyay & A.A.Ansari; Moraceae; QURTUBA 0075	Shah tooth ^P^	King white mulberry, shahtoot mulberry, Tibetan mulberry, or long mulberry	Leaves	Cooked ^P, G^	8	**–**	3
	Kro ^G^						
*Morus nigra* L.; Moraceae; QURTUBA 0076	Toor toth ^P^	Black mulberry	Fruits	Raw snack ^P, G^	22	**–**	26
	Kro ^G^						
*Myrtus communis* L.; Myrtaceae; QURTUBA 0077	Mano ^P^	Myrtle	Fruits	Raw snack ^P^	16	**–**	**–**
*Nasturtium officinale* R.Br.; Brassicaceae; QURTUBA 0078	Chungol ^K^	Watercress	Aerial parts	Cooked ^K, P^	24	22	**–**
	Tarmera ^P^						
*Olea ferruginea* Wall. ex Aitch.; Oleaceae; QURTUBA 0079	Koo ^K^	Indian olive	Fruits	Raw snack ^K, P, G^	28	26	29
	Khuna ^P^						
	Kao ^G^						
*Oxalis corniculata* L.; Oxalidaceae; QURTUBA 0080	Trewaky ^P^	Creeping woodsorrel	Aerial parts	Raw snack ^P^	4	**–**	**–**
*Portulaca oleracea* L.; Portulacaceae; QURTUBA 0081	Lunri ^K^	Common purslane	Aerial parts	Cooked ^K, P, G^	23	26	18
	Warkharay ^P^						
	Lor salori ^G^						
*Papaver rhoeas* L.; Papaveraceae; QURTUBA 0082	Foond ^G^	Common poppy	Shoots	Cooked ^G, K^	**–**	**–**	12
*Pinus gerardiana* Wall. ex D.Don.; Pinaceae; QURTUBA 0083	Shut ^K^		Seeds	Raw snack ^K, G^	**–**	11	17
	Chalghoza ^p^	Chilgoza pine					
*Salvia moorcroftiana* Wall. ex Benth.; Lamiaceae; QURTUBA 0084	Kharwag ^P^	Sage, Kashmir salvia	Fruits and aerial parts	Raw snack ^P, G^	7	**–**	4
	Ghadikan ^G^						
*Piper nigrum* L.; Piperaceae; QURTUBA 0085	Kajmurch ^G^	Black pepper	Fruits	Raw snack ^G, P^	20	**–**	15
	Kali march ^P^						
*Prunus armeniaca* L.; Rosaceae; QURTUBA 0086	Zangali sharay ^p^	Siberian apricot	Fruits	Raw snack ^P, K, G^	13	4	6
	Asher ^K^						
	Hari ^G^						
*Punica granatum* L.; Lythraceae; QURTUBA 0087	Hanar ^K^	Pomegranate	Fruits	Raw snack ^K, P, G^	26	30	29
	Ananghoray ^P^						
	Dharek ^G^						
*Pyrus pashia* Buch.-Ham. ex D.Don.; Rosaceae; QURTUBA 0088	Tangay ^P^	Wild Himalayan pear	Fruits	Raw snack ^K, P, G^	25	23	18
	Tangeer ^K^						
	Tangai ^G^						
*Quercus baloot* Griff.; Fagaceae; QURTUBA 0089	Jhund/bhenaye ^k^	Holly oak	Fruits	Raw snack ^K, P, G^	26	28	22
	Shah balooth ^P^						
	Terleo ^G^						
*Rosa moschata* Hook.f.; Rosaceae; QURTUBA 0090	Kwrach ^P^	Musk rose	Fruits	Raw snack ^K, P^	10	**–**	9
	Falari ^K^						
*Rubus ulmifolius* Schott.; Rosaceae; QURTUBA 0091	Baghanra ^P^	Elmleaf blackberry	Fruits	Raw snack ^P, G^	7	**–**	2
	Bakarun ^G^						
*Rubus fruticosus* Hegetschw.; Rosaceae; QURTUBA 0092	Mangroos ^K^	Bramble	Fruit and leaves	Raw snack and cooked ^K, G^	**–**	13	10
	Groose ^G^						
*Rubus vestitus* Hegetschw.; Rosaceae; QURTUBA 0093	Kharawara ^P^	European blackberry	Fruits	Raw snack ^P^	19	**–**	**–**
*Rumex dentatus* L.; Polygonaceae; QURTUBA 0094	Ubobal ^K^	Toothed dock	Aerial parts	Cooked ^K, P, G^	11	16	13
	Shalkhay ^P^						
	Holo ^G^						
*Rumex hastatus* D. Don.; Polygonaceae; QURTUBA 0095	Cheki ^K^	Heartwing sorrel	Aerial parts	Raw snack ^K, P, G^	10	7	4
	Tarokay ^P^						
	Tarokew ^G^						
*Sageretia thea* (Osbeck) M.C. Johnst.; Rhamnaceae; QURTUBA 0096	Kharo K	Chinese sweet-plum	Seeds	Raw snack ^G, P^	15	8	12
	Gongair ^G^						
	Mamanra ^P^						
*Sophora mollis* (Royle) Graham ex Baker; Fabaceae; QURTUBA 0097	Cheripeer ^G^	Soft sophora	Aerial parts	Cooked ^G^	23	**–**	14
	Marghay khpa ^P^			Raw snack ^P^			
*Sideroxylon mascatense* (A.DC.) T.D.Penn.; Sapotaceae; QURTUBA 0098	Gorgora ^P^	Bully tree	Fruits	Raw snack ^P^	30	**–**	**–**
*Silene conoidea* L.; Caryophyllaceae; QURTUBA 0099	Ladheer ^K^	Large sand catchfly	Fruits	Raw snack ^K, P^	11	2	**–**
	Mangotay ^P^						
*Sisymbrium irio* L.; Brassicaceae; QURTUBA 0100	Jenjar ^P^	London rocket	Seeds	Tea ^P^	2	**–**	5
	Jhenjer ^k^			Raw snack ^G^			
*Solanum nigrum* L.; Solanaceae; QURTUBA 0101	Karmach ^K^	Black nightshade	Fruits	Raw snack ^K, P, G^	11	19	23
	Karmacho ^P^						
	Kachmach ^G^						
*Stellaria media* (L.) Vill.; Caryophyllaceae; QURTUBA 0102	Warghastalay ^P^	Common chickweed	Aerial parts	Cooked ^K, P, G^	5	13	10
	Warghastalay ^P^						
	Stergeo ^G^						
*Trianthema portulacastrum* L.; Aizoaceae; QURTUBA 0103	Chongol ^K^	Desert horse purslane	Aerial parts	Cooked ^K^	**–**	10	**–**
*Trifolium repens* L.; Fabaceae; QURTUBA 0104	Shotal ^P^	White clover	Aerial parts	Cooked ^P, G^	26	10	15
	Shotaleo ^k^						
*Drimia indica* (Roxb.) Jessop.; Asparagaceae; QURTUBA 0105	Jeej ^G^	Indian squill, true squill, or sea onion	Whole plant	Raw snack ^G^	**–**	**–**	18
*Urtica dioica* L.; Urticaceae; QURTUBA 0106	Juon ^K^	Stinging nettle	Leaves	Cooked ^K, P^	6	12	**–**
	Sezonkay ^P^						
*Vicia sativa* L.; Fabaceae; QURTUBA 0107	Arwari ^P^	Common vetch	Fruits	Raw snack ^P^	24	**–**	**–**
*Vitis heyneana* Roem. & Schult.; Vitaceae; QURTUBA 0108	Gedar kwar ^P^	Grape	Shoots	Raw snack ^P, K^	3	4	**–**
	Jnagli loosh ^K^						
*Zanthoxylum armatum* DC.; Rutaceae; QURTUBA 0109	Dambara ^P^	Winged prickly ash	Fruits	Raw snack ^P^	27	**–**	**–**
*Morchella esculenta* (L.) Pers.; Morchellaceae; QURTUBA 0073	Guchi ^G^	Common morel	Whole fruiting bodies	Soup	28	**–**	**–**
*Agaricus campestris* L.; Agaricaceae; QURTUBA 111	Shethi^K^	Meadow mushroom	Whole fruiting bodies	Cooked^K^	**–**	7	**–**

**Fig. 4:**
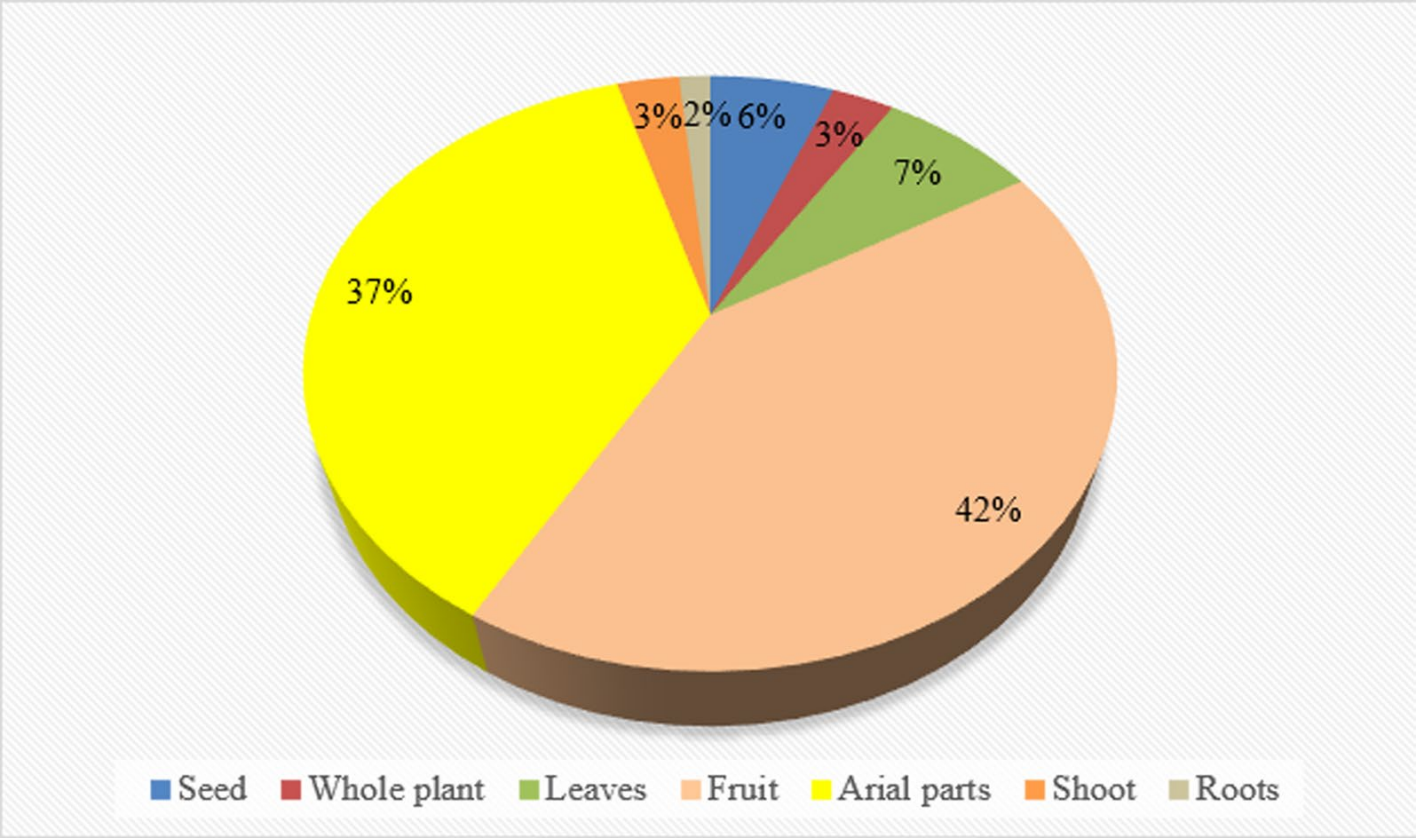
Utilized plant parts of the wild food taxa used in the study area

Our findings revealed that raw snacks were the dominant food category, and it is believed that the emergence or popularity of raw snacks among the various cultural communities might have evolved during mobile pastoralism [8] with local communities co-evolving [10]. Our results are consistent with earlier ethnobotanical studies which frequently found raw snacks to be the main food category [39, 40]. Among the quoted plant taxa, fruits (28 taxa) and aerial parts (25) were the most utilized parts (Fig. 4). The plants which were reported as raw snacks by more than 50% of the participants were nearly all fruits, such as *Berberis lycium*, *Morus alba*, *Celtis tetrandra*, *Morella esculenta*, *Pyrus pashia*, *Olea ferruginea*, *Diospyros lotus*, *Morus nigra*, *Ziziphus oxyphylla*, and *Ziziphus jujuba.* The most commonly reported wild vegetables (50% of the participants) were *Apteranthes tuberculata*, *Rubus fruticosus*, *Chenopodium murale*, *Nasturtium officinale*, *Chenopodium album*, *Sophora mollis*, and *Malva neglecta.* We also observed that some of the taxa that were consumed could have toxic effects, for instance, *Solanum nigrum*, which is locally referred to as *Karmacho*, is known to contain toxic alkaloids [41], mainly found in its fruit [42], but none of the study participants mentioned any toxic effects of these plants, and thus it might be possible that these plants are not consumed in high amounts and as a result do not cause any health issues.

Historically, all three ethnolinguistic groups have frequently practiced pastoralism, and we can therefore assume that this distinctive human–ecological attribute of these communities might have some particular associations with the consumption of specific wild food plant taxa. Research has shown that pastures have been important places for gathering WFPs [11, 15]. Among the reported WFPs, most of the wild vegetables that were gathered by the local groups were agricultural weeds which may have been harvested in anthropogenic environments. Given their role in the food ethnobotanies of the researched communities, we argue that the wide ecological range of weeds [43] may suggest that these taxa constitute an important proportion of the food ethnobotanies of various communities across the globe. As reported by Ahmad et al. [44], various ethnic communities in the North-West Frontier Province of Pakistan frequently use weeds as wild vegetables. The wide cultural acceptability of weeds in local food systems indicates that they could be utilized as an alternative food ingredient, which could play a part in countering food shortages [45–47].

The quoted plants were gathered in different ecological zones; for instance, plants were gathered from mountains, pastures, agricultural lands, along water courses, and from home gardens. Johns et al. [48] claimed that the most frequently quoted WFPs that are widely gathered are those that grow close to human settlements. It has been asserted that the gathering environment plays a special role, which should be the center of focus, rather than looking at the quantity of species harvested [49].

**Fig. 5:**
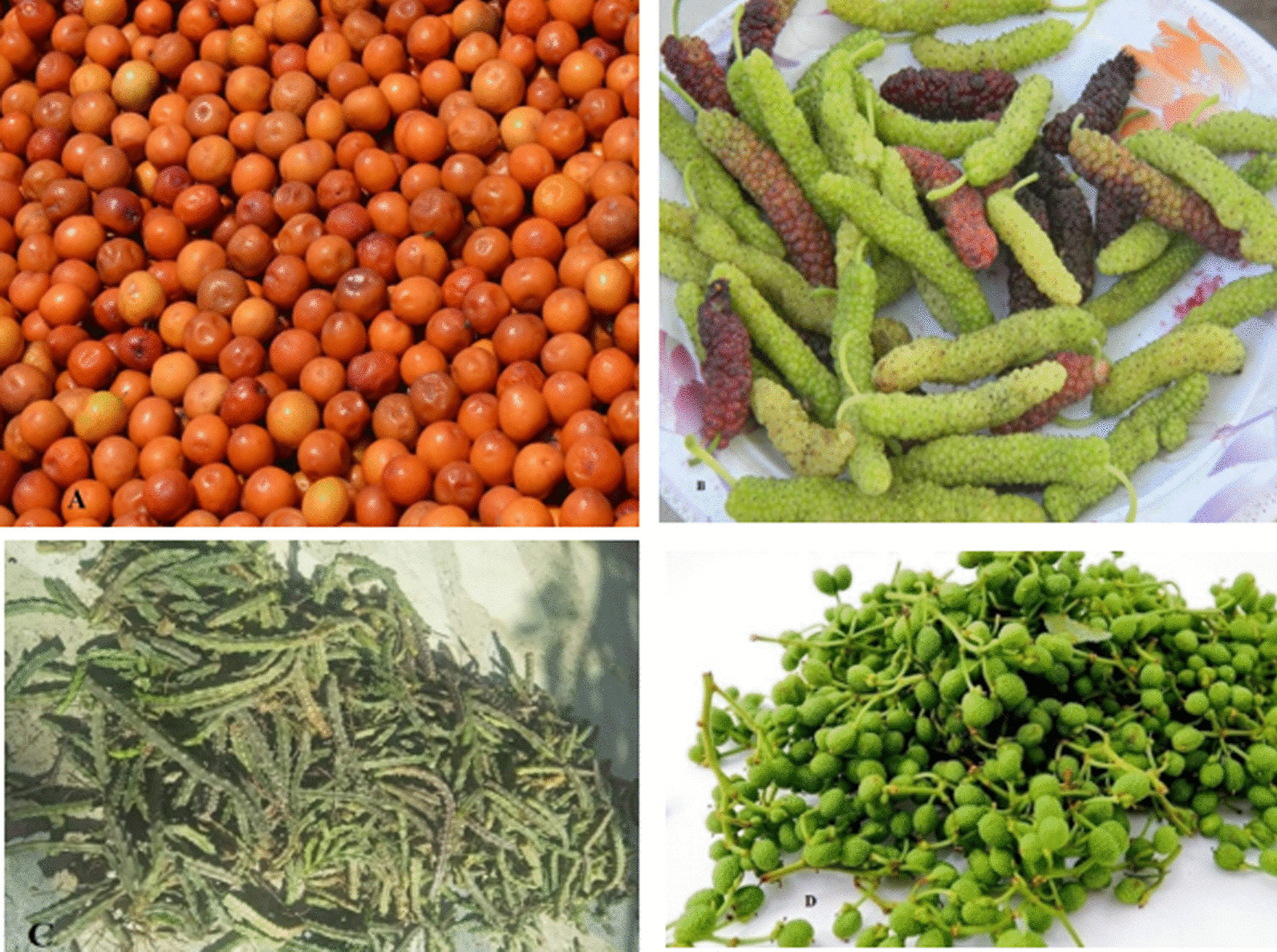
Some examples of wild food plants in Upper Dir District, the Patrak Valley: **a** *Ziziphus oxyphylla*, **b** *Morus macroura* **c** *Apteranthes tuberculata*, **d** *Zanthoxylum armatum*

Some of the most important and common plants that were gathered in the summer season included *Allium carolinianum*, *Medicago polymorpha*, *Apteranthes tuberculata*, and *Zanthoxylum armatum*. We found some WFPs that were gathered and dried for use in different seasons, especially winter, such as *Diospyros lotus*, *Quercus baloot*, and *Pinus gerardiana.* During winter, the kernels of these three plants are consumed on the spot. We have also provided pictorial view of some of the gathered WFPs used in the study area (Fig. 5).

### Cross-cultural comparison

**Fig. 6:**
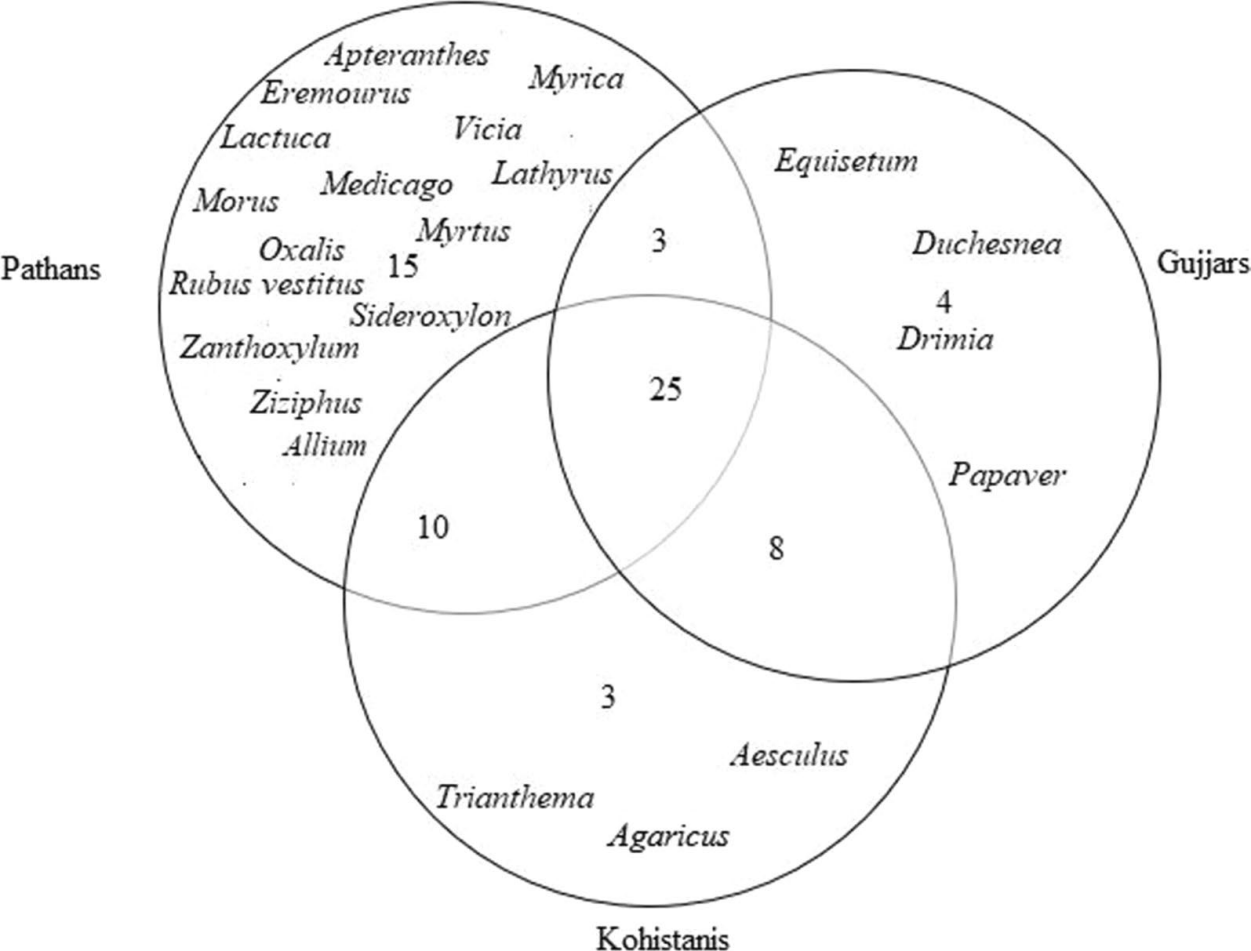
Venn diagram illustrating the overlap of the recorded WFPs among the considered groups

**Fig. 7:**
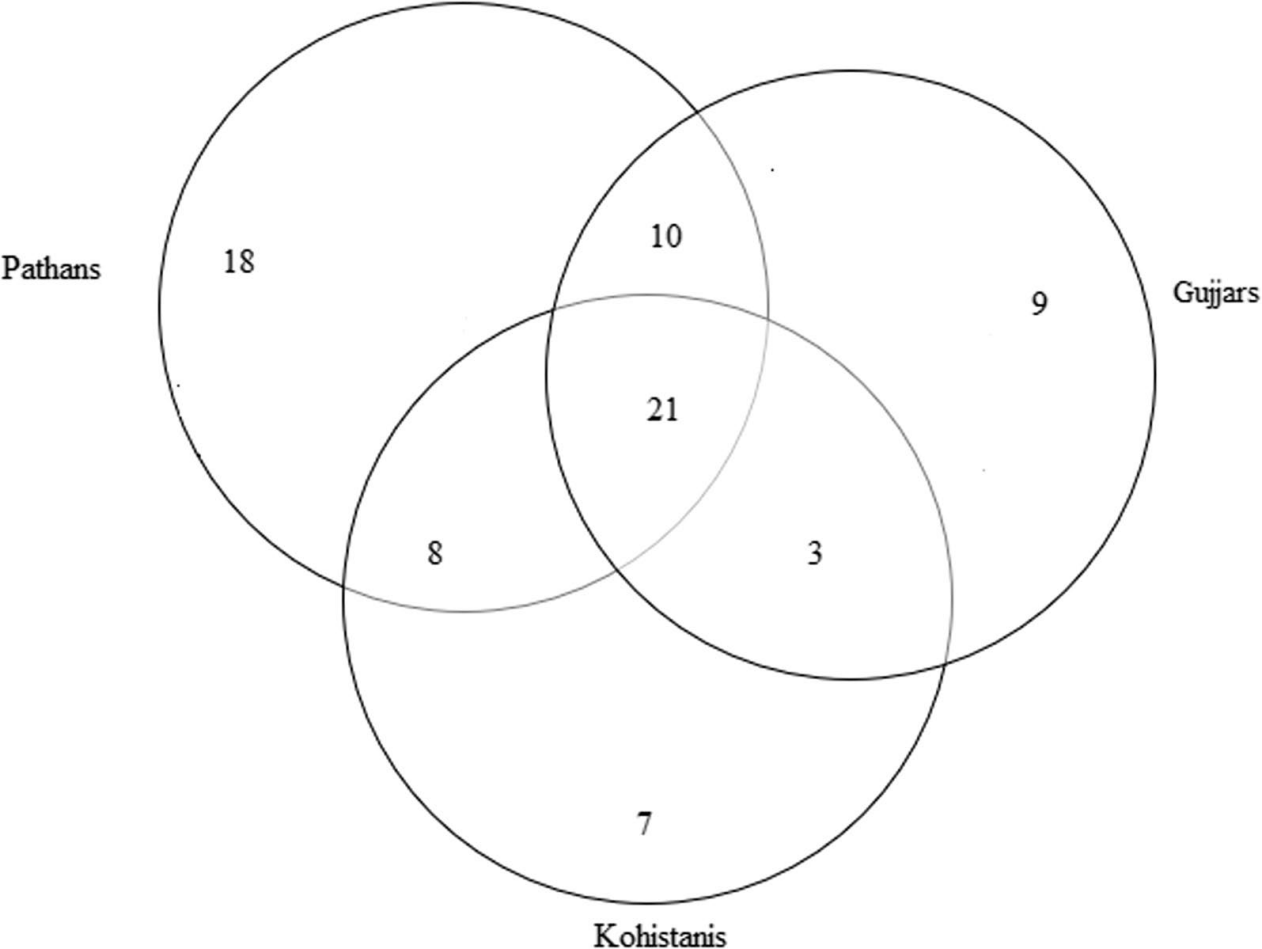
Venn diagram showing the overlap of the WFPs use reports among the three studied groups

Cross-cultural analysis revealed that the food ethnobotanies of the three researched groups are quite heterogeneous (Fig. 6). We found that 37% of the plant uses were commonly reported by all three ethnolinguistic groups, which indicates that the researched communities have diverse knowledge on the reported WFPs. Jaccard indices showed that the greatest number of similarities were among the Gujjar and Kohistani communities (Table 3), while a large number of overlaps were also observed between Pathans and Kohistanis. Pathans retained rich knowledge on WFPs, and they quoted several idiosyncratic uses of the reported plant taxa (Fig. 7). Descriptive data revealed that the greatest number of use reports were quoted by Pathans (1051), followed by that of Gujjars speakers (626) and Kohistanis (588).

**Table 3:** Jaccard similarities indices for all recorded WFPs among the three studied communities

	Pathans	Kohistanis	Gujjars
Pathans	×	0.35	×
Kohistanis	×	×	0.38
Gujjars	0.30	×	×

The overall findings of the current study are quite peculiar which indicates that there could be different factors that have affected the food ethnobotanies of the three studied groups. On the basis of the results of the current investigation, we can affirm that LPK has been equally impacted by both human–ecological attributes and sociocultural drivers. It is worth mentioning that Gujjars are an important ethnic group whose food ethnobotany and related food practices are somewhat distinct from the other two groups (although they have less idiosyncratic uses). Gujjars are nomadic pastoralists and spend most of their time at higher altitudes in mountain pastures across the Hindukush region. The fewer number of taxa as well as the idiosyncratic uses of WFPs among Gujjars might be due to several factors. For instance, Gujjars are quite exclusively pastoralists, while the other two groups have adopted a mixed system of horticulturalism and pastoralism, and thus Gujjars might possess less knowledge on the food uses of agricultural weeds which, in turn, has impacted the total number of plants used in their traditional food system, as it mainly consists of dairy products.

The food ethnobotanical divergences of Gujjars can also be explained by the fact that Gujjars are strictly endogamous and they do not intermarry with the other two ethnic groups, which has limited the diffusion of exotic uses of WFPs into their communities, and thus their knowledge might be more pure and local compared to the other two groups. The comparatively close affinity of Pathans and Kohistanis in terms of plant use also indicates that local plant knowledge has transferred horizontally between the different groups. It has been reported that the close sociocultural interactions among different groups that share the same socio-ecological space also depend on intermarriages between them [15]. The similarities in the food ethnobotanies of Pathans and Kohistanis might also be due to the fact that the Kohistani people have somehow undergone acculturation to the dominant Pathan culture, which in turn has impacted daily cultural practices including local plant knowledge among the minority group. The idiosyncratic uses of plants reported by Gujjars indicate that they have deeper knowledge on plants that grow in higher mountain areas and pastures, and the distinctiveness of their food ethnobotany may also be linked to their identity. Study participants confirmed that Pathan and Kohistani communities often intermarry and thus we suggest that local ecological knowledge on WFPs and the similarities between the two groups are the result of their sociocultural negotiations. Moreover, Pathans and Kohistanis also share the same ecological space within the Patrak Valley.

It is also interesting to note that the local plant nomenclature of the three groups has some commonalities, for instance, *Sisymbrium irio* was known as *Jenjar*, *Rumex hastatus* as *Tarokay*, and *Solanum nigrum* as *Karmacho*, and these three names are locally used by Pathans. These findings show that Gujjars and Kohistanis have also undergone sociolinguistic adaptation in the study area. These two ethnolinguistic groups frequently interact in the local market with Pathans, the dominant cultural group. We have also observed that Pathans have extensive knowledge of the plants recorded among the other ethnic groups, which could be due to their wider access to land and natural resources compared to the other communities. In general, the sociolinguistic adaptation of Gujjars and Kohistanis could be the result of frequent interactions with Pathans, as the latter are often the local healers, herbalists, and timber dealers in the Patrak Valley. This language transition may have also been linked to a more general cultural adjustment by the two minority groups.

### Comparison with the food ethnobotany of Pakistan

On the basis of the comparative analysis between the existing literature on Pakistani (and especially North Pakistani) food ethnobotany and the results of the current study, we can list several notable food uses of plants that are very important to the researched communities. We carefully evaluated and compared the published literature with our research findings. In the recent past, our research group has carried out some important field ethnobotanical studies in North and West Pakistan and has thoroughly collected information on the culinary applications of wild food taxa among different ethnic groups [10, 11, 15, 17, 31–37]. Additionally, we also analyzed some research articles that only partially focused on WFPs and recorded only wild vegetables (a combination of medicinal and food uses). It is important to note that most of these recently conducted studies presented cross-cultural comparisons, which was also an aim of the current study. Comparative analysis revealed that most of the plants have already been documented in earlier food ethnobotanical studies; however, we have still reported some novel food ingredients obtained from certain plant taxa which are new to the Pakistani food ethnobotanical literature. These novel plant ingredients were obtained from *Aesculus indica*, *Agaricus campestris*, *Apteranthes tuberculata*, *Duchesnea indica*, *Equisetum arvense*, *Eremurus himalaicus*, *Eruca vesicaria*, *Isodon rugosus*, *Morella esculenta*, *Sophora mollis*, and *Drimia indica.*

### Food tourism and rural development

**Fig. 8:**
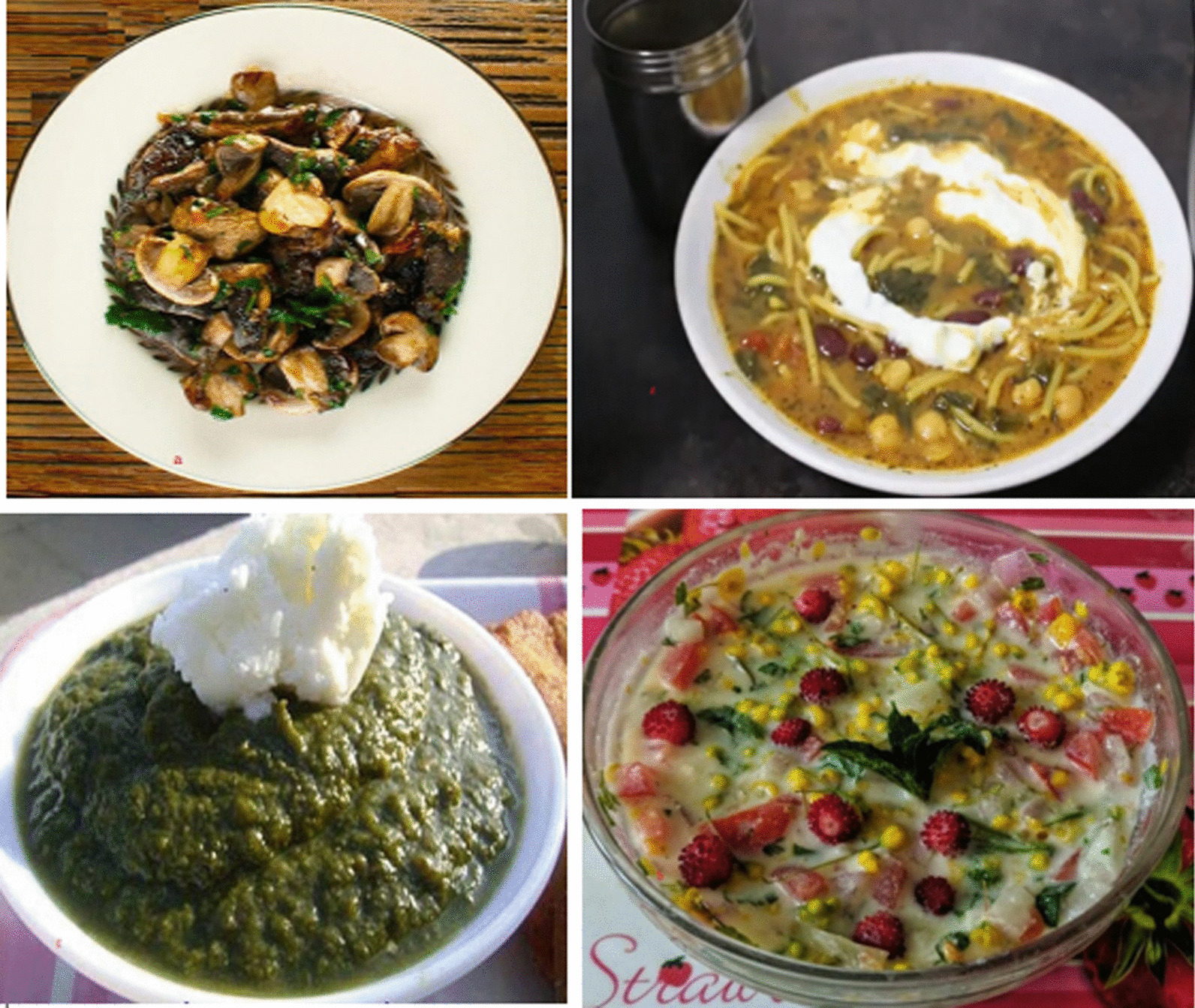
Some of the important local food dishes prepared in the Patrak Valley

We observed that these mountain communities are highly marginalized economically and have very little access to resources. They most often rely on local subsistence activities and the management of small-scale businesses in the area. Looking through the lens of food ethnobotany, we can state that the traditional foods of the researched groups represent a potential source of livelihood if their traditional cultural practices are promoted through ecological tourism. For instance, we found that some of the important foods and drinks that are part of their food heritage have remarkable potential to attract tourists if they are properly encouraged and marketed (see Fig. 8). Here, we provide examples of two foods that are very popular among the three studied groups. In the summer season, Pathans and Gujjars most often use the fruit of *Berberis lycium* to make a recreational drink. The juice of this fruit is extracted and then added to milk before drinking. Similarly, among Gujjars, there is a special soup, mainly prepared during the cold season, made from *Morchella* spp. and *Agaricus* spp. First, onions are chopped into pieces and fried in cooking oil, to which garlic is added. Next, tomato and green chilies are added. Afterward, black pepper, salt, spaghetti noodles, and cumin are mixed together. The green leaves of *Portulaca oleracea* are cut into small pieces and, along with beans, added to the cooking pot. Soon after, *Morella esculenta* and *Agaricus* spp. are cut into small pieces and then, together with white corn flour which has already been mixed with water, added to the pot and cooked. These two examples highlight the food diversity of the studied ethnic groups that could provide a vibrant platform for alleviating the economic instability of these communities.

During the survey, we also observed several plants that were already sold in markets, such as *Apteranthes tuberculata* and *Portulaca oleracea*, the latter a wild vegetable that is extremely well liked in the Upper Dir District of the Patrak Valley. Moreover, we found certain WFPs and mushrooms that the locals had brought down from the highlands, such as *Malva neglecta*, *Allium carolinianum*, and *Morella esculenta*. Locals stated that the gathering of WFPs has been declining gradually, even though some wild vegetables, including *Chenopodium murale*, *Dysphania botrys*, *Allium carolinianum*, and *Eremurus himalaicus*, are still collected and used in the community’s daily food system. We also observed that some of the plants that were used as food or medicine were collected and sold in the market. The Food and Agriculture Organization (FAO) has stated that “wild species and intra-species biodiversity have an important role in world food security” and that “nutrition and biodiversity converge on a shared pathway contributing to food security and economic development” [50]. As the area is a potential habitat of many medicinal and food plants, local communities may benefit from the commercial food and medicinal ingredients derived from these plants if their collection is systematized and institutionalized. We have confirmed that some of the plants have very high demand, and thus their widespread harvest comes at the expense of the survival of certain taxa in the study area. We suggest strong political activism regarding the sustainable harvest of wild food plants in this region in order to obtain long-term and health benefits. A sustainable harvest will be equally beneficial for both socio-economic sustainability and environmental sustainability. Overgrazing is also a problem that could be solved through the mutual understanding of relevant stakeholders via economic incentives. Research has shown that many areas in Hindukush regions have experienced a significant loss in ecosystem functions as a result of a variety of socioeconomic and biophysical factors that have contributed to the depletion of natural resources [51].

### Revitalization of local plant knowledge

Local plant knowledge has been an integral part of daily life, and it plays a pivotal role in sustaining human life on this planet. During our previous research, one of the major problems we have encountered in different parts of the Hindukush, even in remote mountain communities, is that local plant knowledge is slowly eroding or being replaced by exotic knowledge. Modern agricultural practices are spreading across mountain areas, and as a result, traditional ecological practices are disappearing, which might have significant negative effects on local food sovereignty and security in the future. Moreover, we have observed that in the face of socioenvironmental change, local foraging practices are gradually disappearing among many mountain societies, and thus ethnobotanical studies devoted to recording the disappearing knowledge on wild plants could represent a remarkable resource to fight future calamities.

We argue that local ecological practices are crucial for countering the impacts of future food scarcity as it solves the problem from inside the community instead of from outside. Mountain areas have fragile physical and geographical characteristics, and food mobility is a major problem in times of environmental hardship, and therefore we need a complementary system which can equally endorse the sustainable use of cultivated and wild food resources. Wild food supplies certainly cannot meet demand, but without them the gap between food availability and need would be much greater than expected in the future.

In order to ensure the better use of local natural resources, we need to educate our youth as they are the real inheritors of cultural heritage, and they can play a greater role in promoting awareness of local sustainable practices. As has been observed, traditional knowledge on plants has been gradually decreasing as a result of social change and people are more reliant on commodified plant ingredients, thus posing a threat to the sustainability of LPK. Abbas et al. [36] stated that the traditional/local ecological knowledge (LEK) of WFPs in West Pakistan has been lost to some extent, and only around one-third of the informants were able to name most of the reported species. However, LEK is partially still present in the memories and practices of local residents, for example, in the areas surrounding the Hindukush Mountains in North Pakistan [17]. In order to create WFP-centered approaches, however, policies on food security and biodiversity protection must be revised.

Our research group has found some important strategies for revitalizing LEK in schools, which could provide a foundation for promoting these practices in other mountain territories. For example, together with the local community in the Yasin Valley of Pakistan, we identified some approaches that could help in the revitalization of LEK among young community members, such as study trips, traditional food day celebrations, developing WFP herbaria, art competitions, and the introduction of “food scouting” (ethnobiology-centered documentation of threatened local foods) into school curricula [52].

### Ecological transition and food security

Biodiversity underpins our economic, cultural, and social well-being; however, man-made changes to ecosystems have been more rapid in the past 50 years than at any time in human history. Today, around one million species of an estimated eight million animal and plant species are already threatened with extinction. The situation is critical as more countries around the world are already experiencing the impacts of climate change—from longer periods of drought to more and stronger storms, heat waves, and wildfires. In the current changing climate conditions, ecological systems are disrupted and change rapidly, which is directly connected to biodiversity loss and ecological degradation.

The wild food plants that make up a prominent proportion of the food baskets of the local food system among many human societies are also under threat. Local plant habitats, even in very remote areas in different parts of the globe, have often been impacted by global changes. Plant availability has seriously decreased in different geographical contexts due to manmade activities such as intense agricultural practices, population expansion, armed conflicts, overharvesting, uncontrolled grazing. For instance, in some conflict/war zones in North-West Pakistan, the growth and availability of WFPs is seriously reduced in the proximity of agricultural fields. Among other issues, monoculture-centered farming practices have a very negative impact on soil fertility that in turn comes at the expense of wild flora. In Pakistani Hindukush, there has already been a significant degradation of natural resources [51] and it can be expected that in the near future these ecological disruptions will present a great challenge for the food security of local communities, especially the most marginalized ones.

In the current debate on ecological transition, it is urgent that policy makers articulate appropriate measures to avoid devastating future food crises. Food sovereignty-centered and place-centered food resources, sociabilities, and even activisms will have to play an important role in promoting sustainable ecological practices and a prudent use of local natural food resources. Despite the fact that many political stakeholders around the globe are committed to taking strong precautionary measures to mitigate unsustainable practices, there is still a huge gap between policy and practicality of the recommended strategies on the ground, and therefore WFP-centered practices urgently need to be revitalized and shaped by local communities and ethnobiologists working together.

## Conclusions

The study recorded remarkable knowledge on WFPs among three different ethnic communities in the highlands of the Hindukush. The study also revealed that each of the communities has retained distinct food ethnobotanies. The Gujjar community showed comparatively less affinity in terms of WFP uses with the other two groups. The idiosyncrasy nature of the recorded data indicates that human ecological attributes have played a central role in the gathering of WFPs. Similarly, sociocultural communication has been vital in transmitting LEK among the studied communities.

In the wave of socio-environmental change, LEK could be lost, which could have negative impacts on food security in the near future. Appropriate strategies should be adopted by policy makers to integrate LEK into future development infrastructure. In addition, local ecological knowledge should be considered when designing food-related policies. Likewise, food activism should be encouraged and the protection of local plant knowledge should be incentivized.

Moreover, to alleviate the economic marginalization of poor households, food tourism could represent a better option for promoting traditional food products. We also suggest that future ethnographic studies focus more on Gujjars, who live in different parts of the Hindukush and practice mobile pastoralism, in order to document and celebrate their relevant wild food-related environmental practices and adopt better policies for sustaining their human–ecological system.

## Data Availability

All the data are available in this article.
